# Crystal structure of the co-crystal butyl­paraben–isonicotinamide (1/1)

**DOI:** 10.1107/S2056989015023518

**Published:** 2016-01-01

**Authors:** Rajni M. Bhardwaj, Huaiyu Yang, Alastair J. Florence

**Affiliations:** a Strathclyde Institute of Pharmacy and Biomedical Sciences, University of Strathclyde, 161 Cathedral Street, Glasgow G4 0RE, Scotland

**Keywords:** crystal structure, butyl­paraben, isonicotinamide, co-crystal, hydrogen bonding

## Abstract

The title 1:1 co-crystal, C_11_H_14_O_3_·C_6_H_6_N_2_O [systematic name: butyl 4-hy­droxy­benzoate–isonicotinamide (1/1)], crystallizes with one mol­ecule of butyl­paraben (BPN) and one mol­ecule of isonicotinamide (ISN) in the asymmetric unit. In the crystal, BPN and ISN mol­ecules form hydrogen-bonded (O—H⋯N and N—H⋯O) dimers of paired BPN and ISN mol­ecules. These dimers are further connected to each other *via* N—H⋯O=C hydrogen bonds, creating ribbons in [011] which further stack along the *a* axis to form a layered structure with short C⋯C contacts of 3.285 (3) Å. Packing inter­actions within the crystal structure were assessed using PIXEL calculations.

## Abstract 2

The title 1:1 co-crystal, butyl­paraben–isonicotinamide [BPIN, butyl 4-hy­droxy­benzoate isonicotinamide (1/1)], crystallizes with one mol­ecule each of butyl­paraben and isonicotinamide. In the crystal, various BPN and ISN mol­ecules are linked *via* O—H⋯N, N—H⋯O and N—H⋯O=C hydrogen bonds, creating a layered structure.

## Chemical context   

Butyl­paraben (butyl 4-hy­droxy­benzoate, BPN), a naturally derived preservative, is widely used in pharmaceutical products and cosmetics (Charnock & Finsrud, 2007 ▸), and generally considered to be safe (Hossaini *et al.*, 2000 ▸). The solubility of BPN has been reported in various solvents (Yang & Rasmuson, 2010 ▸; 2012 ▸; 2013 ▸). Isonicotinamide (ISN) is a widely used coformer (Aakeröy *et al.*, 2003 ▸) and is known to form hydrogen-bonded co-crystals with phenolic compounds (Vishweshwar *et al.*, 2003 ▸; McKellar *et al.*, 2014 ▸). The sample of butyl paraben–isonicotinamide (BPIN) co-crystals was isolated during an experimental co-crystal screening of BPN. The sample was identified as a novel form using multi-sample foil transmission X-ray powder diffraction analysis (Florence *et al.*, 2003 ▸). A suitable sample for single crystal X-ray diffraction analysis was obtained from slow evaporation of 1:1 molar solution of BPN with ISN in ethanol at room temperature.
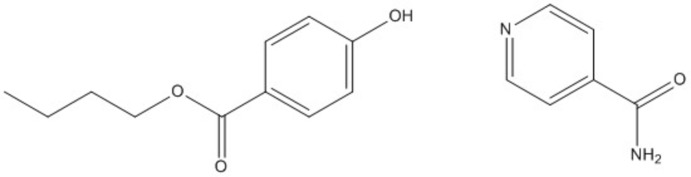


## Structural commentary   

**Figure 1:**
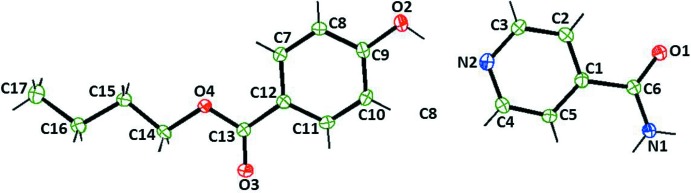
A view of the mol­ecular structure of the asymmetric unit of the title co-crystal, showing the atom labelling. Displacement ellipsoids are drawn at the 50% probability level.

The title co-crystal crystallizes with one mol­ecule of BPN and a mol­ecule of ISN in the asymmetric unit (Fig. 1 ▸). In the solid state, the BPN mol­ecule exhibits a planar conformation with a fully extended *trans* zigzag butyl ester group.

## Supra­molecular features   

**Figure 2:**
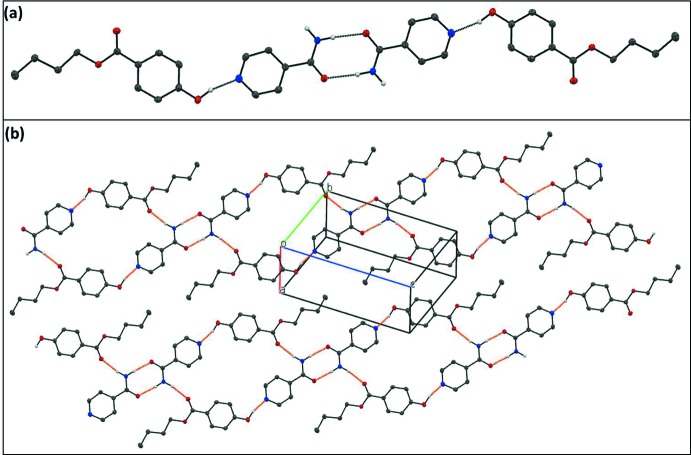
Hydrogen bonds in the title compound: (*a*) hydrogen-bonded (thin grey lines) dimer of paired BPN⋯ISN mol­ecules; (*b*) hydrogen-bonded (thin orange lines) ribbon of dimers extended in [011]. Atom colour code: C, N, O and H are grey, blue, red and white, respectively. Hydrogen atoms not involved in hydrogen bonding have been omitted for clarity.

**Figure 3:**
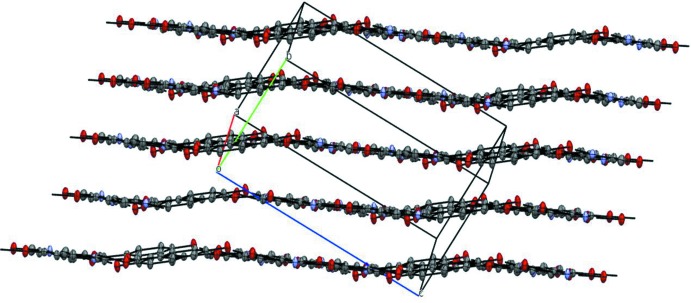
A portion of the crystal packing showing the layered structure of the title co-crystal. H atoms have been omitted for clarity.

The crystal structure is defined by hydrogen-bonded BPN–ISN–ISN–BPN dimers of paired BPN⋯ISN mol­ecules connected *via* O—H⋯N hydrogen bonds (Fig. 2 ▸ *a*). These BPN–ISN–ISN–BPN dimers are further connected to each other *via* N—H⋯O=C hydrogen-bonds extending the structure to form ribbons in [011]; see Fig. 2 ▸ *b* and Table 1 ▸. These ribbons further stack along *a* axis to produce a layered structure (Fig. 3 ▸) which is stabilized by various van der Waals inter­actions and exhibits short C⋯C contacts of 3.285 (3) Å. PIXEL (Gavezzotti, 2002 ▸; 2003 ▸) calculations revealed that the largest contribution to crystal stabilization comes from the dispersion energy (E_d_, −98.5 kJ mol^−1^). The next greatest contribution comes from electrostatic (Coulombic) energy, (E_C_, −67.3 kJ mol^−1^) and then from polarization energy (E_p_, −32.2 kJ mol^−1^).

## Database survey   

The crystal structures of BPN (CSD refcode: UDOMIL) (Yang & Rasmuson, 2013 ▸) and its clathrate hydrate (CSD refcode: VOFKIL) have been reported in the literature (de Vries & Caira, 2008 ▸). In UDOMIL, the BPN mol­ecule exhibits a planar conformation except for the terminal ethyl moiety of butyl ester group which is in a *cis* orientation with respect to the ester group.

## Synthesis and crystallization   

Plate shaped crystals were grown from the saturated 1:1 molar solution of BPN with ISN in ethanol by isothermal solvent evaporation at 298 K.

## Refinement   

Crystal data, data collection and structure refinement details are summarized in Table 2 ▸. The N and O bound H atoms were located in a difference Fourier map and isotropically refined. The C-bound H atoms were placed in calculated positions and refined as riding atoms: C—H = 0.95–0.99 Å with *U*_iso_(H) = 1.5*U*_eq_(C) for methyl H atoms and = 1.2*U*_eq_(C) for other H atoms.

## Supplementary Material

Crystal structure: contains datablock(s) I. DOI: 10.1107/S2056989015023518/cv5494sup1.cif

Structure factors: contains datablock(s) I. DOI: 10.1107/S2056989015023518/cv5494Isup2.hkl

Supporting information file. DOI: 10.1107/S2056989015023518/cv5494Isup3.cml

CCDC reference: 1440986

Additional supporting information:  crystallographic information; 3D view; checkCIF report

## Figures and Tables

**Table 1:** Hydrogen-bond geometry (Å, °)

*D*—H⋯*A*	*D*—H	H⋯*A*	*D*⋯*A*	*D*—H⋯*A*
O2—H3*O*⋯N2	0.95 (3)	1.79 (3)	2.721 (2)	165 (2)
N1—H1*N*⋯O1^i^	0.91 (2)	1.97 (2)	2.880 (2)	175.3 (15)
N1—H2*N*⋯O3^ii^	0.94 (2)	2.02 (2)	2.948 (2)	168.3 (18)

Symmetry codes: (i) 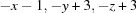; (ii) 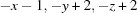.

**Table 2:** Experimental details

Crystal data	
Chemical formula	C_11_H_14_O_3_·C_6_H_6_N_2_O
*M*_r_	316.35
Crystal system, space group	Triclinic, *P* 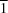
Temperature (K)	150
*a*, *b*, *c* (Å)	5.6257 (6), 9.8661 (11), 14.3979 (15)
α, β, γ (°)	90.834 (7), 91.431 (7), 91.645 (7)
*V* (Å^3^)	798.47 (15)
*Z*	2
Radiation type	Mo *K*α
μ (mm^−1^)	0.09
Crystal size (mm)	0.45 × 0.36 × 0.21

Data collection	
Diffractometer	Bruker APEXII CCD
Absorption correction	Multi-scan (*SADABS*; Bruker, 2007 ▸)
*T*_min_, *T*_max_	0.625, 0.745
No. of measured, independent and observed [*I* > 2σ(*I*)] reflections	10444, 3225, 2344
*R*_int_	0.039
(sin θ/λ)_max_ (Å^−1^)	0.627

Refinement	
*R*[*F* ^2^ > 2σ(*F* ^2^)], *wR*(*F* ^2^), *S*	0.045, 0.116, 1.05
No. of reflections	3225
No. of parameters	221
H-atom treatment	H atoms treated by a mixture of independent and constrained refinement
Δρ_max_, Δρ_min_ (e Å^−3^)	0.26, −0.27

Computer programs: *APEX2* and *SAINT* (Bruker, 2007 ▸), *SHELXS97* (Sheldrick, 2008 ▸), *SHELXL2014* (Sheldrick, 2015 ▸), *Mercury* (Macrae *et al.*, 2008 ▸), *ORTEP-3 for Windows* (Farrugia, 2012 ▸), *enCIFer* (Allen *et al.*, 2004 ▸) and *publCIF* (Westrip, 2010 ▸).

## supplementary crystallographic information

### Crystal data

**Table:**

C_11_H_14_O_3_·C_6_H_6_N_2_O	*Z* = 2
*M**_r_* = 316.35	*F*(000) = 336
Triclinic, *P*1	*D*_x_ = 1.316 Mg m^−^^3^
*a* = 5.6257 (6) Å	Mo *K*α radiation, λ = 0.71073 Å
*b* = 9.8661 (11) Å	Cell parameters from 4038 reflections
*c* = 14.3979 (15) Å	θ = 2.5–26.4°
α = 90.834 (7)°	µ = 0.09 mm^−^^1^
β = 91.431 (7)°	*T* = 150 K
γ = 91.645 (7)°	Plate, colourless
*V* = 798.47 (15) Å^3^	0.45 × 0.36 × 0.21 mm

### Data collection

**Table:**

Bruker APEXII CCD diffractometer	3225 independent reflections
Radiation source: fine-focus sealed tube	2344 reflections with *I* > 2σ(*I*)
Graphite monochromator	*R*_int_ = 0.039
φ and ω scans	θ_max_ = 26.5°, θ_min_ = 2.1°
Absorption correction: multi-scan (*SADABS*; Bruker, 2007)	*h* = −6→7
*T*_min_ = 0.625, *T*_max_ = 0.745	*k* = −12→12
10444 measured reflections	*l* = −18→18

### Refinement

**Table:**

Refinement on *F*^2^	0 restraints
Least-squares matrix: full	Hydrogen site location: mixed
*R*[*F*^2^ > 2σ(*F*^2^)] = 0.045	H atoms treated by a mixture of independent and constrained refinement
*wR*(*F*^2^) = 0.116	*w* = 1/[σ^2^(*F*_o_^2^) + (0.0482*P*)^2^ + 0.3682*P*] where *P* = (*F*_o_^2^ + 2*F*_c_^2^)/3
*S* = 1.05	(Δ/σ)_max_ = 0.001
3225 reflections	Δρ_max_ = 0.26 e Å^−^^3^
221 parameters	Δρ_min_ = −0.27 e Å^−^^3^

### Special details

**Table:**

Geometry. All e.s.d.'s (except the e.s.d. in the dihedral angle between two l.s. planes) are estimated using the full covariance matrix. The cell e.s.d.'s are taken into account individually in the estimation of e.s.d.'s in distances, angles and torsion angles; correlations between e.s.d.'s in cell parameters are only used when they are defined by crystal symmetry. An approximate (isotropic) treatment of cell e.s.d.'s is used for estimating e.s.d.'s involving l.s. planes.

### Fractional atomic coordinates and isotropic or equivalent isotropic displacement parameters (Å^2^)

**Table:**

	*x*	*y*	*z*	*U*_iso_*/*U*_eq_	
H1N	−0.623 (3)	1.480 (2)	1.4226 (13)	0.019 (5)*	
H2N	−0.626 (4)	1.399 (2)	1.3256 (16)	0.035 (6)*	
H3O	0.140 (5)	0.939 (3)	1.1807 (17)	0.054 (7)*	
O4	0.1197 (2)	0.47988 (13)	0.82145 (8)	0.0227 (3)	
O1	−0.2641 (2)	1.38157 (14)	1.49057 (9)	0.0275 (3)	
N1	−0.5594 (3)	1.41555 (17)	1.38514 (11)	0.0238 (4)	
O2	0.2637 (2)	0.88155 (15)	1.16227 (9)	0.0308 (4)	
N2	−0.0392 (3)	1.05682 (16)	1.24155 (10)	0.0234 (4)	
O3	−0.2134 (2)	0.59634 (14)	0.80166 (9)	0.0297 (3)	
C13	−0.0315 (3)	0.57543 (19)	0.84578 (12)	0.0207 (4)	
C7	0.2633 (3)	0.63083 (19)	0.97609 (12)	0.0220 (4)	
H8	0.3619	0.5641	0.9542	0.026*	
C6	−0.3631 (3)	1.35566 (18)	1.41440 (12)	0.0194 (4)	
C12	0.0462 (3)	0.65243 (18)	0.93023 (12)	0.0190 (4)	
C14	0.0586 (3)	0.40447 (19)	0.73615 (12)	0.0223 (4)	
H15A	0.0438	0.4660	0.6845	0.027*	
H15B	−0.0918	0.3552	0.7423	0.027*	
C8	0.3330 (3)	0.7076 (2)	1.05364 (13)	0.0239 (4)	
H9	0.4775	0.6920	1.0839	0.029*	
C2	−0.0520 (3)	1.18936 (19)	1.38204 (13)	0.0234 (4)	
H2	0.0162	1.2121	1.4399	0.028*	
C10	−0.0302 (3)	0.83018 (19)	1.04128 (12)	0.0229 (4)	
H11	−0.1290	0.8969	1.0631	0.027*	
C16	0.2264 (3)	0.2420 (2)	0.62252 (13)	0.0242 (4)	
H17A	0.0722	0.1955	0.6171	0.029*	
H17B	0.2306	0.3126	0.5764	0.029*	
C5	−0.3533 (3)	1.21153 (19)	1.26476 (12)	0.0213 (4)	
H6	−0.4918	1.2498	1.2420	0.026*	
C15	0.2548 (3)	0.30708 (19)	0.71924 (12)	0.0220 (4)	
H16A	0.2511	0.2370	0.7658	0.026*	
H16B	0.4075	0.3551	0.7248	0.026*	
C1	−0.2580 (3)	1.25031 (18)	1.35125 (12)	0.0185 (4)	
C9	0.1866 (3)	0.80852 (19)	1.08652 (12)	0.0221 (4)	
C11	−0.0986 (3)	0.75279 (19)	0.96404 (12)	0.0223 (4)	
H12	−0.2437	0.7680	0.9342	0.027*	
C3	0.0504 (3)	1.0945 (2)	1.32551 (13)	0.0251 (4)	
H3	0.1888	1.0544	1.3467	0.030*	
C4	−0.2387 (3)	1.11486 (19)	1.21283 (13)	0.0240 (4)	
H5	−0.3042	1.0892	1.1551	0.029*	
C17	0.4194 (4)	0.1414 (2)	0.60224 (14)	0.0314 (5)	
H18A	0.5725	0.1869	0.6068	0.047*	
H18B	0.3946	0.1042	0.5407	0.047*	
H18C	0.4130	0.0696	0.6465	0.047*	

### Atomic displacement parameters (Å^2^)

**Table:**

	*U*^11^	*U*^22^	*U*^33^	*U*^12^	*U*^13^	*U*^23^
O4	0.0233 (7)	0.0256 (7)	0.0192 (7)	0.0096 (6)	−0.0068 (5)	−0.0082 (5)
O1	0.0308 (7)	0.0313 (8)	0.0203 (7)	0.0134 (6)	−0.0072 (6)	−0.0097 (6)
N1	0.0260 (9)	0.0268 (9)	0.0185 (8)	0.0106 (7)	−0.0047 (7)	−0.0089 (7)
O2	0.0272 (7)	0.0367 (8)	0.0279 (7)	0.0099 (6)	−0.0072 (6)	−0.0183 (6)
N2	0.0251 (8)	0.0230 (8)	0.0223 (8)	0.0055 (7)	0.0000 (6)	−0.0049 (7)
O3	0.0284 (7)	0.0376 (8)	0.0229 (7)	0.0145 (6)	−0.0098 (6)	−0.0089 (6)
C13	0.0215 (9)	0.0228 (10)	0.0181 (9)	0.0066 (8)	−0.0013 (7)	−0.0014 (8)
C7	0.0227 (9)	0.0230 (10)	0.0205 (9)	0.0081 (8)	−0.0021 (7)	−0.0043 (8)
C6	0.0209 (9)	0.0199 (9)	0.0173 (9)	0.0022 (8)	−0.0009 (7)	−0.0019 (7)
C12	0.0211 (9)	0.0195 (9)	0.0164 (9)	0.0033 (7)	−0.0010 (7)	−0.0006 (7)
C14	0.0257 (10)	0.0252 (10)	0.0157 (9)	0.0049 (8)	−0.0061 (7)	−0.0061 (8)
C8	0.0190 (9)	0.0311 (11)	0.0215 (9)	0.0076 (8)	−0.0060 (7)	−0.0061 (8)
C2	0.0234 (9)	0.0270 (10)	0.0195 (9)	0.0037 (8)	−0.0046 (7)	−0.0050 (8)
C10	0.0226 (9)	0.0241 (10)	0.0222 (9)	0.0090 (8)	0.0014 (7)	−0.0035 (8)
C16	0.0281 (10)	0.0246 (10)	0.0199 (9)	0.0060 (8)	−0.0034 (8)	−0.0053 (8)
C5	0.0234 (9)	0.0230 (10)	0.0176 (9)	0.0068 (8)	−0.0040 (7)	−0.0013 (8)
C15	0.0233 (9)	0.0232 (10)	0.0194 (9)	0.0062 (8)	−0.0042 (7)	−0.0027 (8)
C1	0.0200 (9)	0.0176 (9)	0.0179 (9)	0.0017 (7)	0.0009 (7)	−0.0015 (7)
C9	0.0239 (10)	0.0239 (10)	0.0184 (9)	0.0031 (8)	−0.0009 (7)	−0.0067 (8)
C11	0.0196 (9)	0.0276 (10)	0.0199 (9)	0.0079 (8)	−0.0037 (7)	−0.0010 (8)
C3	0.0225 (9)	0.0263 (10)	0.0265 (10)	0.0095 (8)	−0.0033 (8)	−0.0059 (8)
C4	0.0277 (10)	0.0269 (11)	0.0173 (9)	0.0055 (8)	−0.0029 (7)	−0.0058 (8)
C17	0.0348 (11)	0.0310 (11)	0.0287 (11)	0.0096 (9)	0.0016 (9)	−0.0062 (9)

### Geometric parameters (Å, º)

**Table:**

O4—C13	1.336 (2)	C12—C11	1.391 (2)
O4—C14	1.455 (2)	C14—C15	1.506 (2)
O1—C6	1.238 (2)	C8—C9	1.395 (2)
N1—C6	1.330 (2)	C2—C3	1.379 (2)
O2—C9	1.354 (2)	C2—C1	1.387 (2)
N2—C4	1.334 (2)	C10—C11	1.380 (3)
N2—C3	1.341 (2)	C10—C9	1.392 (3)
O3—C13	1.215 (2)	C16—C17	1.523 (3)
C13—C12	1.475 (2)	C16—C15	1.528 (2)
C7—C8	1.382 (3)	C5—C4	1.387 (2)
C7—C12	1.397 (2)	C5—C1	1.388 (2)
C6—C1	1.514 (2)		
			
C13—O4—C14	115.95 (13)	C3—C2—C1	118.97 (17)
C4—N2—C3	117.20 (15)	C11—C10—C9	120.08 (16)
O3—C13—O4	122.73 (16)	C17—C16—C15	112.76 (15)
O3—C13—C12	123.99 (16)	C4—C5—C1	118.94 (16)
O4—C13—C12	113.28 (14)	C14—C15—C16	110.66 (15)
C8—C7—C12	120.73 (16)	C2—C1—C5	118.09 (16)
O1—C6—N1	123.13 (16)	C2—C1—C6	117.54 (16)
O1—C6—C1	118.87 (15)	C5—C1—C6	124.37 (15)
N1—C6—C1	117.99 (16)	O2—C9—C10	122.75 (16)
C11—C12—C7	118.73 (16)	O2—C9—C8	117.70 (16)
C11—C12—C13	118.87 (15)	C10—C9—C8	119.55 (16)
C7—C12—C13	122.38 (15)	C10—C11—C12	120.95 (17)
O4—C14—C15	107.55 (14)	N2—C3—C2	123.48 (17)
C7—C8—C9	119.96 (17)	N2—C4—C5	123.31 (17)
			
C14—O4—C13—O3	2.3 (3)	O1—C6—C1—C2	−1.0 (3)
C14—O4—C13—C12	−177.25 (15)	N1—C6—C1—C2	179.41 (18)
C8—C7—C12—C11	0.0 (3)	O1—C6—C1—C5	179.42 (18)
C8—C7—C12—C13	178.19 (18)	N1—C6—C1—C5	−0.2 (3)
O3—C13—C12—C11	2.0 (3)	C11—C10—C9—O2	179.70 (18)
O4—C13—C12—C11	−178.52 (16)	C11—C10—C9—C8	−0.4 (3)
O3—C13—C12—C7	−176.20 (19)	C7—C8—C9—O2	−179.54 (17)
O4—C13—C12—C7	3.3 (3)	C7—C8—C9—C10	0.6 (3)
C13—O4—C14—C15	177.62 (16)	C9—C10—C11—C12	0.1 (3)
C12—C7—C8—C9	−0.4 (3)	C7—C12—C11—C10	0.1 (3)
O4—C14—C15—C16	−170.00 (15)	C13—C12—C11—C10	−178.09 (17)
C17—C16—C15—C14	−179.45 (17)	C4—N2—C3—C2	−0.4 (3)
C3—C2—C1—C5	0.6 (3)	C1—C2—C3—N2	−0.2 (3)
C3—C2—C1—C6	−179.01 (17)	C3—N2—C4—C5	0.7 (3)
C4—C5—C1—C2	−0.4 (3)	C1—C5—C4—N2	−0.3 (3)
C4—C5—C1—C6	179.21 (17)		

### Hydrogen-bond geometry (Å, º)

**Table:**

*D*—H···*A*	*D*—H	H···*A*	*D*···*A*	*D*—H···*A*
O2—H3*O*···N2	0.95 (3)	1.79 (3)	2.721 (2)	165 (2)
N1—H1*N*···O1^i^	0.91 (2)	1.97 (2)	2.880 (2)	175.3 (15)
N1—H2*N*···O3^ii^	0.94 (2)	2.02 (2)	2.948 (2)	168.3 (18)

Symmetry codes: (i) −*x*−1, −*y*+3, −*z*+3; (ii) −*x*−1, −*y*+2, −*z*+2.
